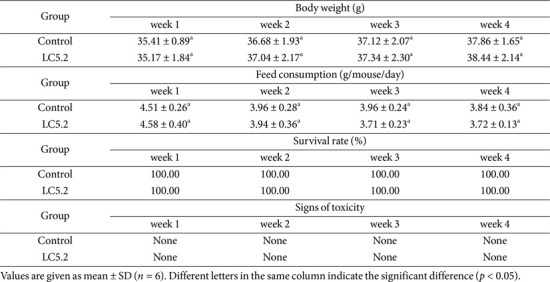# Characterization and Evaluation of *Lactobacillus plantarum* LC5.2 Isolated from Thai Native Pigs for its Probiotic Potential in Gut Microbiota Modulation and Immune Enhancement

**DOI:** 10.4014/jmb.2503.03028

**Published:** 2025-06-17

**Authors:** Kittiya Khongkool, Malai Taweechotipatr, Sunchai Payungporn, Vorthon Sawaswong, Monthon Lertworapreecha

**Affiliations:** 1 Biotechnology program, Faculty of Science and Digital Innovation, Thaksin University, Phatthalung Province 93210, Thailand; 2 Center of Excellence in Probiotic Research, Faculty of Medicine, Srinakharinwirot University, Bangkok 10110, Thailand; 3 Center of Excellence in Systems Microbiology, Faculty of Medicine, Chulalongkorn University, Pathumwan, Bangkok 10330, Thailand; 4 Department of Biochemistry, Faculty of Science, Mahidol University, Bangkok 10400, Thailand; 5 Microbial Technology for Agriculture, Food, and Environment Research Center, Faculty of Science and Digital Innovation, Thaksin University, Phatthalung Province 93210, Thailand

**Keywords:** *Lactobacillus plantarum*, probiotic properties, gut microbiota modulation, Thai native pigs, safety assessment

## Abstract

Probiotic supplementation, particularly with *Lactobacillus* species, enhances growth performance, maintains gastrointestinal microbial balance, and prevents infections in livestock. This study isolated *Lactobacillus* strains from the feces of healthy native pigs in southern Thailand and assessed their probiotic properties and safety through both *in vitro* and *in vivo* evaluations. Nine *Lactobacillus* strains showed probiotic potential, with *Lactobacillus plantarum* LC5.2 demonstrating the best characteristics. This strain tolerated both acid and bile (100% tolerance) and exhibited strong adhesion properties, including high auto-aggregation (69.74%), cell surface hydrophobicity (77.14%), adhesion to Caco-2 cells (9.31%), and biofilm formation. It also exhibited antibacterial activity, inhibiting EHEC, EPEC, and *Salmonella* Typhimurium through organic acid production. Co-aggregation with these pathogens ranged from 60.83% to 74.09%. Safety evaluations showed no hemolytic activity, susceptibility to antibiotics, and co-existence with other probiotics. In mice, *L. plantarum* LC5.2 showed no toxicity, with normal food intake, behavior, and weight gain. No abnormalities were found in the small intestine, colon, liver, or spleen. Mice administered the probiotic had significantly higher intestinal IgA levels. Gut microbiome analysis revealed no notable structural alterations but indicated an increase in beneficial bacteria, including *Lactobacillus*. These results suggest that *L. plantarum* LC5.2 demonstrates strong probiotic potential, safety, and benefits for gut health, making it a promising candidate for livestock applications.

## Introduction

Antibiotics have played a pivotal role in managing infectious diseases in livestock, improving animal growth and overall health. However, the excessive and prolonged use of antibiotics in animal husbandry has led to the emergence of antibiotic-resistant bacteria, which can be transmitted to humans through contaminated meat and other pathways, raising significant public health concerns. In response, the World Health Organization (WHO) has strongly recommended reducing antibiotic use in livestock, particularly for growth promotion and disease prevention without proper diagnosis [1].

This shift in regulatory guidelines presents a considerable challenge for livestock farmers, necessitating the exploration of alternative methods to maintain animal health. A promising strategy is the supplementation of probiotics in animal feed, which helps promote gut health and reduces reliance on antibiotics. Probiotics are live microorganisms that, when administered in adequate amounts, confer health benefits to the host [2]. They enhance gut microbiota composition, improve digestion, boost nutrient absorption, and prevent infections. Additionally, probiotics modulate immune responses, stimulate immune cells, and inhibit pathogen adhesion, leading to improved overall health and immunity in animals. Probiotics also regulate inflammatory responses, maintaining gut balance and reducing pathogen colonization [3].

However, not all bacterial strains exhibit strong probiotic properties. The effectiveness of probiotics can be species-specific and is often enhanced when the strain is isolated from the same animal species [4]. Probiotics used in livestock are typically sourced from the gastrointestinal tract or feces of the same species [5]. Effective probiotics must meet essential criteria, such as stress tolerance, adhesion capability, antipathogenic activity, and safety [6]. Although commercial probiotic products are becoming more widely available, common issues such as mislabeling of species, contamination, and insufficient viable cell counts still persist, which may reduce their intended health benefits. [7]. Consequently, there is a continuous need to identify and develop new probiotic strains with suitable properties for specific animal species and geographic regions.

This study involved the isolation of *Lactobacillus* strains from the feces of healthy native pigs in southern Thailand, followed by an evaluation of their probiotic properties and safety for potential applications in livestock management.

## Materials and Methods

### Sample Collection and Lactic Acid Bacteria Isolation

A total of seventy-one fecal samples were collected from healthy native pigs raised in backyard environments across the southern provinces of Thailand, including Phatthalung, Nakorn Si Thammarat, and Krabi. These pigs, approximately 5-6 months old at the time of sample collection, were raised in small-scale backyard settings and fed a diet of locally grown plants and leftovers. Fresh fecal samples were obtained early in the morning after cleaning the pig pens, with only the central portion collected using a sterile spoon to minimize contamination. The samples were then stored in sterile tubes, transported in iceboxes, and analyzed promptly upon arrival. For lactic acid bacteria isolation, the method followed the procedure described in a previous study [8], with minor modifications. One gram of each fecal sample was homogenized with 9 ml of sterile normal saline solution (0.85% w/v NaCl) for 5 min. The samples were then serially diluted, and 0.1 ml of the appropriate dilution was spread on De Mann, Rogosa, and Sharpe (MRS) agar (Hi-media, India) and incubated anaerobically at 37°C for 48 h. After incubation, 5–10 single colonies with typical lactic acid bacteria characteristics from each sample were selected and purified. Preliminary identification of each isolate was based on Gram staining, catalase reaction, and cell morphology. Isolates presumed to be *Lactobacillus* were identified as Gram-positive, catalase-negative, and rod-shaped. These isolates were preserved in MRS broth (Hi-media) with 20% glycerol and stored at −80°C for further studies.

### Screening of Antipathogenic Activity

The antipathogenic activity of the isolates was determined using the agar-well diffusion method described in a previous study [8] with minor modifications. In this study, three pathogenic bacteria were selected as indicators, including enterohemorrhagic *Escherichia coli* (EHEC) strain SC2451-1, enteropathogenic *E. coli* (EPEC) strain SC2451-2, and *Salmonella enterica* ser. Typhimurium (*S*. Typhimurium) strain SC2451-3. Each *Lactobacillus* isolate was cultured in MRS broth and incubated anaerobically at 37°C for 48 h. After incubation, cell-free culture supernatants (CFCS) were obtained by centrifugation at 5,000 ×*g* for 10 min. Concurrently, the pathogenic strains were cultured in Tryptic Soy Broth (TSB) (Hi-media, Mumbai, India) at 37°C for 24 h. Pathogenic cells were collected, washed, and adjusted to a 0.5 McFarland standard (1.5 × 10^8^ CFU/ml) using a suspension turbidity detector (Den-1B, BioSan, Latvia) in sterile normal saline solution. Then, 100 μl of each adjusted pathogenic strain was spread onto Nutrient Agar (NA) plates (Hi-media). Six-millimeter wells were made using a borer. Subsequently, 80 μl of CFCS, both untreated and pH-adjusted to 7.0, was added to the wells. The plates were incubated at 37°C for 15 h, and the diameter of the clear zone around each well, indicating antibacterial activity, was measured after incubation.

### Acid and Bile Tolerance Tests

Acid and bile salt tolerance assays were performed as described previously [9] with minor modifications. Overnight cultures of *Lactobacillus* were harvested, washed, and adjusted to a 0.5 McFarland standard in sterile saline. For the acid tolerance assay, 1 ml of the cell suspension was inoculated into MRS broth, which was adjusted to pH 3 using 1 M HCl. For the bile salt tolerance assay, 1 ml of the suspension was inoculated into MRS broth containing 1% (w/v) bile salt (Hi-media). Both assays were incubated at 37°C, and viable colony counts were assessed at 0 and 3 h using the drop plate technique [10]. Tolerance was evaluated by comparing the colony counts at 3 h to the initial counts at 0 h.

### Auto-Aggregation Assays

Auto-aggregation was assessed as described previously [11], with minor modifications. *Lactobacillus* isolates were cultured overnight in MRS broth and collected by centrifugation at 5,000 ×*g* for 10 min. The cells were washed and resuspended in sterile phosphate buffered saline (PBS) to an optical density (OD) of 0.8 ± 0.2 at 600 nm. Subsequently, 5 ml suspension was incubated at 37°C without agitation. At 2, 4, 8, and 24 h, 200 μl of the upper suspension was collected, and OD at 600 nm was measured using a microplate reader (Biochrom Asys UVM 340 Scanning Microplate Reader, England) with PBS as the blank. The percentage of auto-aggregation was calculated using the following equation:

$$\text{Auto-aggregation}\left(\text{\%}\right)\text{=}\left[\text{1-}\left({\text{A}}_{\text{t}}{\text{÷A}}_{\text{0}}\right)\right]\text{×100}$$

where A_t_ is the absorbance at different times (2, 4, 8 or 24 h) and A_0_ is the initial absorbance value. Auto-aggregation percentages were classified as low (16–35%), intermediate (35–50%), and high (>50%) [12].

### Cell Surface Hydrophobicity

Cell surface hydrophobicity was assessed using the bacterial adhesion to hydrocarbon (BATH) test with xylene as the hydrocarbon, following previously established protocols [13, 14], with slight modifications. Overnight cultures of each isolate were harvested, washed, and adjusted to an OD of 0.6 – 0.8 at 600 nm using PBS. Next, 1 ml of xylene was vigorously mixed with 3 ml of the bacterial suspension for 2 min and allowed to stand undisturbed at 37°C for 1 h. After phase separation, 200 μl of the aqueous phase was carefully removed for OD measurement at 600 nm. The cell surface hydrophobicity was calculated as a percentage using the following equation:

$$\text{Cell surface hydrophobicity (\%)=}\left[\frac{\left({\text{A}}_{\text{0}}\text{-A}\right)}{{\text{A}}_{\text{0}}}\right]\text{×100}$$

where A_0_ is the absorbance before mixing and A is the absorbance after mixing. Based on their adhesion to hydrocarbons, bacterial strains are categorized into strong hydrophobic (>50%), moderate hydrophobic (20%–50%), and low hydrophobic (<20%) [15].

### Biofilm Formation

Biofilm formation was assessed using the microtiter dish biofilm formation assay, as previously described [16]. To assess biofilm formation, overnight cultures of each *Lactobacillus* isolate were collected, washed, and adjusted to a 0.5 McFarland standard using sterile normal saline solution. This cell suspension was diluted 1:100 in MRS broth supplemented with 5% glucose, and 100 μl was added to each well of a 96-well plate for anaerobic incubation at 37°C for 72 h. For staining of biofilms, the planktonic cells were removed, and the wells were washed twice with 200 μl of sterile distilled water before being dried at room temperature for 24 h. The biofilm was stained for 10 min using 125 μl of a 0.1% crystal violet solution. The crystal violet solution was then removed, and the wells were washed twice with 200 μl of distilled water and dried at room temperature overnight. To quantify the biofilm, 150 μl of 95% ethanol was added to each well and incubated at room temperature for 10 min. The solubilized crystal violet was transferred to a new 96-well plate, and the OD was measured at 570 nm. Bacterial adherence was classified based on mean OD values: <0.120 indicated non-adherence and weak biofilm formation, 0.120–0.240 indicated moderate adherence and moderate biofilm formation, and >0.240 indicated strong adherence and high biofilm formation [17].

### Adhesion to Caco-2 Cells

Caco-2 cells (ATCC HTB-37, Lot: 70032505) were purchased from the American Type Culture Collection (ATCC, USA). The cells were cultured in cell culture bottles with Dulbecco’s Modified Eagle’s Medium (DMEM; Invitrogen, Thermo Fisher Scientific, USA), supplemented with 10% (v/v) heat-inactivated fetal bovine serum (Invitrogen, Thermo Fisher Scientific) and 100 U/ml penicillin along with 100 μg/ml streptomycin. The culture medium was regularly replaced, and the cells were subcultured upon reaching 80-90% confluency.

The adhesion assay followed method described previously [18], with slight modifications. The Caco-2 cells were sub-cultured at 2×10^5^ cells/ml in 24-well plates and grown at 37°C with 5% CO_2_ for 21 days to promote differentiation. The medium was replaced with fresh medium every 2 days and with fresh non-supplemented DMEM at least 1 h before the adhesion assay. Overnight cultures of *Lactobacillus* isolates were harvested, washed twice with PBS, and suspended to a final concentration of 1 × 10^8^ CFU/ml in DMEM, with initial bacterial counts confirmed by plate counting. The confluent cells were washed twice with PBS, after which 1 ml of bacterial suspension was added to each well of the tissue culture plate and incubated at 37°C in 5% CO_2_ for 2 h. After co-incubation, the medium was carefully removed, and each well was washed twice with PBS to remove unbound bacteria. Then, 1 ml of Triton X-100 solution (0.1% v/v in PBS) was added to each well and incubated at room temperature for 10 min to detach the bound bacteria from the Caco-2 cells. The cell lysates were serially diluted, plated on MRS agar, and incubated anaerobically at 37°C for 48 h. The number of adherent cells was determined by the plate count method. The percentage of adhesion was calculated by comparing the viable colony counts of adhered bacteria to the total number of added bacteria.

### Co-Aggregation with Pathogens

Co-aggregation was evaluated following a method described previously [11]. Each isolate was tested for co-aggregation with three pathogenic strains, including EHEC strain SC2451-1, EPEC strain SC2451-2 and *S*. Typhimurium strain SC2451-3. Overnight cultures of the *Lactobacillus* isolates and pathogenic strains were harvested, washed, and resuspended in PBS to an OD of 0.6 – 0.8 at 600 nm. Equal volumes (2 ml) of each *Lactobacillus* isolate and pathogenic strain were thoroughly mixed and incubated at 37°C for 4 h without agitation. After incubation, the upper supernatant was carefully collected for OD measurement at 600 nm. The co-aggregation was calculated using equation:

$$\text{Co-aggregation (\%)=100×}\left[\frac{\left({\text{OD}}_{\text{Lacto}}{\text{+OD}}_{\text{Pathogen}}\right)\text{-2}\left({\text{OD}}_{\text{mix}}\right)}{\left({\text{OD}}_{\text{Lacto}}{\text{+OD}}_{\text{Pathogen}}\right)}\right]$$

where OD_Lacto_ and OD_pathogen_ are absorbances in control tubes containing only *Lactobacillus* isolates or the pathogenic strain, respectively, and OD_mix_ is the absorbance of the mixed suspension at 4 h.

### Hemolytic Activity Tests

The fresh culture of each *Lactobacillus* isolate was streaked on a sheep blood agar plate (ready-to-use culture media, M and P IMPEX, Thailand) and incubated at 37°C for 24 h. After incubation, the hemolytic activity was recorded by observing a hydrolysis zone around the colonies: β-hemolysis (clear zones around colonies), α-hemolysis (green-hued zones around colonies) and γ-hemolysis (no zones around colonies) [19].

### Antibiotic Susceptibility Tests

Antibiogram was studied by disk diffusion assays. Seven antibiotics (Himedia) were tested including ampicillin (25 μg), cephalothin (30 μg), chloramphenicol (30 μg), gentamicin (120 μg), streptomycin (10 μg), tetracycline (10 μg), and vancomycin (30 μg). A 100 μl aliquot of each *Lactobacillus* isolate, adjusted to a 0.5 McFarland standard, was spread onto MRS agar plates and left to dry at room temperature for 5 min. Subsequently, antibiotic discs were placed on the surface of the agar plates and incubated for 18 ± 2 h at 35 ± 1°C. After incubation, the diameters of the inhibition zones, including the diameter of the discs, were measured. The criteria for judgment were based on a previous study [20].

### Co-Existence Assays

The co-existence of tested isolates was examined by a cross-streak method described previously [9], with slight modifications. This test verifies that individual strains do not inhibit one another in a multi-strain application. Each fresh culture was adjusted to 0.5 McFarland, and a loopful of each isolate was streaked perpendicularly on MRS agar plates. Antagonistic interactions were assessed after anaerobic incubation at 37°C for 48 h.

### Species Identification by 16S rRNA Gene Sequencing

Genomic DNA was extracted from pure bacterial cultures using the GF-1 Bacterial DNA Extraction Kit (Malaysia) according to the manufacturer’s protocol. The DNA concentration and purity were assessed with a NanoDrop Lite Spectrophotometer (Thermo Fisher Scientific). PCR amplification was performed using universal primers 27F (5’-AGAGTTTGATCCTGGCTCAG-3’) and 1492R (5’-GGTTACCTTGTTACGACTT-3’) as described in a previously described method [21]. PCR was performed in a 50 μl reaction using a thermocycler (MultiGeneTM Mini Personal Cycler, USA) with an initial activation at 95°C for 3 min, followed by 30 cycles of 95°C for 1 min, 55°C for 1 min, and 72°C for 1 min, with a final extension at 72°C for 5 min. The PCR products, approximately 1,466 bp, were analyzed by agarose gel electrophoresis and purified using the PCR Clean-up kit (GF-1 AmbiClean Kit (Gel and PCR), Vivantis Technologies, Malaysia). The purified products were then sent for nucleotide sequence analysis at 1st BASE DNA Sequencing Services (Base-Asia, Singapore). Sequences were aligned using Molecular Evolutionary Genetic Analysis (MEGA) software version 11 and compared to representative sequences in the National Center for Biotechnology Information (NCBI) database via Basic Local Alignment Search Tool (BLAST). The *Lactobacillus* gene sequences were submitted to NCBI for accession numbers and the probiotic strain was deposited at the Thailand Bioresource Research Center (TBRC).

### Species-Specific Detection by Multiplex Polymerase Chain Reaction (PCR)

To definitively identify isolates of the *Lactobacillus plantarum* group, a multiplex species-specific PCR assay was performed following a previously described method [22]. The assay utilized recA gene-based primers: paraF (5’-GTCACAGGCATTACGAAAAC-3’), pentF (5’-CAGTGGCGCGGTTGATATC-3’), planF (5’-CCGTTTATG CGGAACACCTA-3’), and pREV (5’-TCGGGATTACCAA ACATCAC-3’). PCR amplification was carried out with the following cycling conditions: initial denaturation at 94°C for 3 min, followed by 30 cycles of 95°C for 30 sec, 56°C for 10 sec, and 72°C for 30 sec, with a final extension at 72°C for 5 min. PCR products were visualized by agarose gel electrophoresis, where 318 bp amplicons indicated *Lactobacillus plantarum*, 218 bp for *Lactobacillus pentosus*, and 107 bp for *L. paraplantarum*.

### Ethics Statement

All animal procedures were conducted in accordance with the guidelines for the care and use of laboratory animals and were approved by the Animal Ethics Committee of Srinakharinwirot University, under approval number COA/AE-003-2566.

### Animals and Experimental Design

*In vivo* studies were conducted using *L. plantarum* LC5.2, identified as the most promising probiotic candidate in the *in vitro* evaluation. Male *Mus musculus* (ICR) mice, aged 6-7 weeks and specific-pathogen-free, were obtained from the National Laboratory Animal Center at Mahidol University (NLAC-MU), Thailand. The mice were acclimatized for one week in groups of three in each cage, under a 12-h light/dark cycle at 24 ± 1°C. The animals were randomly assigned into two groups, with six mice in each group, housed three mice in each cage. During the 30-day study, both groups were given a basal diet (082G/15, NLAC-MU, Mahidol University) and *ad libitum* access to water, which was changed every 2 day. The probiotic group received 100 μl of *L. plantarum* LC5.2 (1 × 10^11^ CFU/ml) daily by oral gavage, while the control group received 100 μl of sterile PBS.

### Monitoring and Observations of Oral Toxicity

Mice were monitored for toxicity signs following the methods in a previous study [23], including changes in skin, fur, eyes, mucous membranes, respiratory and motor activity, behavior, tremors, convulsions, salivation, diarrhea, lethargy, sleep patterns, and gait. Physical parameters such as body weight, feed intake, injuries, and mortality rates were recorded throughout the 30-day study, with daily observations. Body weights were measured weekly, and daily feed residues were assessed to calculate average feed consumption per animal.

### Measurement of Total Secretory Immunoglobulin A (sIgA) in Intestinal Fluids

To investigate the secretory IgA levels in the intestinal fluids, the intestinal fluid was collected by the removal of the small intestine from the gastro-duodenal region to the ileocecal junction and rinsing it with 2 ml of sterile PBS. The fluid from each mouse was then centrifuged at 5,000 ×*g* for 10 min at 4°C, and the supernatant was filtered through sterile 0.22 μm syringe filters. Following this collection process, the secretory IgA levels in the intestinal fluids were measured using the IgA Mouse Uncoated ELISA Kit (Invitrogen, Thermo Fisher Scientific). A microplate was coated with anti-mouse IgA monoclonal antibody and incubated overnight at 4°C. After incubation, the plate was washed, blocked with blocking buffer, and then incubated at room temperature for 2 h, followed by two washes with wash buffer. Each sample or standard was then added to the appropriate wells and incubated at room temperature for 2 h. After washing the plate four times, HRP-conjugated anti-mouse IgA polyclonal antibody was added to each well and incubated at room temperature for 1 h, after which the plate was washed four more times. Subsequently, TMB substrate solution was added to each well and incubated at room temperature for 15 min. The reaction was terminated by adding 100 μl of 2 N H_2_SO_4_ to each well, and absorbance was measured at 450 nm for data analysis.

### Histopathological Analysis of Internal Organs

The internal organs of the mice were collected for histopathological analysis according to standard protocols [24]. Mice were fasted overnight before necropsy for clinical pathology. After anesthetization with isoflurane, the mice were humanely euthanized and placed on a sterile dissection bench. A midline incision was made, extending from the navel to the mouth, with additional cuts downward and laterally. The skin, muscle layers, and abdominal membrane were removed to expose the internal organs. The small intestine, colon, liver, and spleen were identified, separated, and placed in individual petri dishes with sterile phosphate-buffered saline to remove blood and contaminants.

Histological assessment was conducted following the standard procedures established by Department of Pathology at the Faculty of Medicine, Chulalongkorn University, Thailand. Organs were fixed in 4% paraformaldehyde phosphate buffer for 24 h. Tissue samples were dehydrated through sequential ethanol solutions (70%, 80%, 95%, and 100% for 1 h each) and cleared with xylene using the Leica TP1020 Tissue Processor (Leica Microsystems, USA). Afterward, the samples were infiltrated with paraffin wax using the Modular Paraffin Embedding Center (MEDITE TES Valida, USA) and allowed to solidify. Thin sections measuring 4-6 μm in thickness were subsequently cut with a microtome (HistoCore MULTICUT-Semi-Automated Rotary Microtome, Leica Microsystems), flattened on a warm water bath, mounted on glass slides, deparaffinized with xylene, rehydrated, and stained with hematoxylin and eosin. Finally, the stained sections were dehydrated, cleared, and mounted with coverslips for examination under a light microscope (Olympus UC50, Japan).

### Analysis of Gut Microbiome Composition

After euthanasia, the colon was cut and the fecal pellets were gently extracted using sterile forceps, then placed into a sterile tube containing DNA/RNA shield reagent and mixed thoroughly. Total DNA was extracted using a ZymoBIOMICS DNA Miniprep Kit (Zymo Research, USA) according to the manufacturer's protocol. The preparation and sequencing of the full-length 16S library were carried out according to the protocol outlined in a previous study [25]. Initially, the full-length 16S rDNA (V1-V9 regions) of bacteria was amplified via PCR using universal primers (27F/1492R) that included 5’ nanopore adaptor tails. After amplification, DNA library from each sample was incorporated with the multiplexing Nanopore barcodes using the PCR Barcoding Expansion 1–96 (EXP-PBC096) kit, followed by purification and quantification. The DNA samples were then pooled in equal amounts, purified, and used for adaptor ligation using the Ligation Sequencing Kit (SQK-LSK112, Oxford Nanopore Technologies, UK) following the standard protocol. The final adaptor-ligated DNA library was loaded and sequenced on the R10.4 (Q20+) flow cell using the MinION Mk1C sequencer (Oxford Nanopore Technologies).

For bioinformatic analysis, the raw FAST5 data from sequencing was basecalled using the guppy basecaller in super-accuracy (SUP) mode. Quality checks of the FASTQ sequences were performed using MinIONQC, followed by demultiplexing and adaptor trimming with Porechop. The reads were processed with NanoCLUST for clustering, polishing, and taxonomic identification. The resulting taxonomic data and abundance information were analyzed using MicrobiomeAnalyst [26].

### Statistical Analysis

All experiments were conducted in triplicate, with the results presented as the average of three independent experiments, and variations indicated by standard deviation. Statistical analysis was carried out using one-way ANOVA (IBM SPSS Statistics 22, SPSS Inc., USA). Tukey's HSD test was applied to compare the mean values of probiotic properties across bacterial strains. An independent t-test was used to compare the mean sIgA levels between the control and probiotic groups. Statistical significance was considered at *p*<0.05.

## Results

### Isolation of Lactic Acid Bacteria from Native Pig Feces

A total of 590 bacterial isolates, presumptively identified as *Lactobacillus* spp., were isolated from 71 fecal samples collected from healthy native pigs. All isolates exhibited typical characteristics of lactic acid bacteria, including Gram-positive staining, catalase negativity, and rod-shaped morphology.

### Antibacterial Activity Against Pathogenic Bacteria

The CFCS from 590 isolates were tested for antibacterial activity using the agar-well diffusion method. A total of 325 isolates (55.08%) showed inhibitory activity against EHEC SC2451-1, 293 isolates (49.66%) inhibited EPEC SC2451-2, and 265 isolates (44.92%) inhibited *S*. Typhimurium SC2451-3. After neutralization of the CFCS to pH 7, no antibacterial activity was observed in any of the isolates (data not shown).

### Acid Tolerance Ability

From the antibacterial activity assay, only isolates that inhibited all three tested pathogens were selected for acid tolerance testing. Isolates that exhibited at least 80% survival after 3 h of exposure to pH 3 were selected for further evaluation. A total of nine *Lactobacillus* isolates satisfied the selection criteria. Among them, LC4.2 and LC5.2 displayed the highest acid tolerance, with survival rates of 113.76% and 100.00%, respectively. The remaining isolates showed survival rates ranging from 80.21% to 90.47% (Table 1).

### Bile Tolerance Ability

The nine acid-tolerant *Lactobacillus* isolates were subsequently tested for bile tolerance. All nine isolates demonstrated strong bile resistance, with survival rates ranging from 89% to 139% (Table 1). Among them, LC4.2, LC5.6, LC5.1, and LC5.2 showed exceptional bile tolerance, with survival rates of 139.01%, 115.18%, 111.50%, and 100.00%, respectively. The remaining isolates also exhibited strong bile tolerance, with survival rates between 89%and 98%.

### Auto-Aggregation Ability

The auto-aggregation ability of the nine *Lactobacillus* isolates was evaluated, and the results are shown in Table 2. All isolates demonstrated a gradual increase in auto-aggregation over time. Isolate LC5.2 exhibited significantly higher auto-aggregation compared to the other isolates, particularly during the first 2 to 8 h of incubation. After 24 h, the auto-aggregation rates of all isolates ranged from 48.98% to 79.14%, with isolate LC5.1 displaying a moderate rate of 48.98% ± 0.79. Among them, LC4.2 showed the highest rate of 79.14% ± 0.76, while LC5.2 maintained a relatively high aggregation rate of 69.74% ± 0.58. Statistically significant differences were observed among the isolates (*p* < 0.05).

### Cell Surface Hydrophobicity

The cell surface hydrophobicity of the nine *Lactobacillus* isolates was evaluated using the BATH test with xylene as the hydrocarbon, and the results are summarized in Table 2. The isolates exhibited varying levels of hydrophobicity, which were classified into three categories. The first group, demonstrating strong hydrophobicity (>50%), included LS5.6, LC1.1, LC5.2, and LF5.2. The second group, with moderate hydrophobicity (20 –50%), consisted of LF5.3 and LS8.7. The third group, exhibiting low hydrophobicity (<20%), comprised LC4.2, LC5.1, and LC5.6. Statistical analysis revealed significant differences in hydrophobicity among the isolates (*p* < 0.05).

### Biofilm Formation

The biofilm-forming ability of the *Lactobacillus* isolates was evaluated using crystal violet retention, with the OD570 values shown in Table 2. All nine isolates were capable of biofilm formation, with OD values between 0.14 and 0.24, indicating moderate adherence. Among them, LC5.2 and LF5.2 demonstrated the highest biofilm production, each with an OD570 value of 0.24.

### Adhesion to Caco-2 Cells

Caco-2 cells were used as an intestinal epithelial model to evaluate bacterial adhesion, focusing on specific ligand–receptor interactions. Plate count analysis revealed that approximately 1 × 10^6^ CFU/ml adhered from an initial 1 × 10^8^ CFU/ml. All isolates demonstrated successful adhesion, with adherence rates ranging from 2.17% to 10.15% (Table 2). Notably, LF5.2, LC5.2, and LC1.1 exhibited the highest adhesion rates, at 10.15 ± 0.42%, 9.31 ± 1.32%, and 9.00 ± 0.41%, respectively.

### Co-Aggregation Ability

Co-aggregation ability of the *Lactobacillus* isolates with EHEC strain SC2451-1, EPEC strain SC2451-2, and *S*. Typhimurium strain SC2451-3 was assessed, with the results shown in Table 3. All isolates exhibited the highest co-aggregation with EHEC (approximately 62-74%), followed by *S*. Typhimurium (approximately 53-64%) and EPEC (approximately 48-61%). Among these, LC5.2 demonstrated the highest co-aggregation, with values of 74.09 ± 0.68% for EHEC, 64.37 ± 0.39% for *S*. Typhimurium, and 60.83 ± 1.04% for EPEC.

### Hemolytic Activity and Co-existence Among *Lactobacillus* Isolates

None of the isolates exhibited β-hemolysis, while most displayed α-hemolysis. Only one isolate, LC5.2, demonstrated γ-hemolysis (Table 4). Regarding co-existence among *Lactobacillus* isolates, no antagonistic interactions were observed.

### Antibiotic Susceptibility Profiles

Antibiotic susceptibility of the *Lactobacillus* isolates was assessed using disk diffusion assays, with the results summarized in Table 4. All isolates were susceptible to ampicillin, chloramphenicol, gentamicin, and streptomycin. However, they showed resistance to vancomycin and tetracycline. The susceptibility to cephalothin varied among the isolates.

### Species Identification

The species of the nine *Lactobacillus* isolates were identified through 16S rRNA gene sequencing, with BLAST results showing that eight isolates exhibited high similarity (>98%) to *L. plantarum*, while LC5.2 was closely related to *L. pentosus* (Table 5). Due to the high similarity between *L. plantarum*, *L. pentosus*, and *L. paraplantarum*, a multiplex species-specific PCR assay targeting a conserved region of the recA gene was subsequently conducted to ensure precise identification. The results confirmed that all isolates were *L. plantarum*, as indicated by the presence of a 318 bp PCR product.

### Oral Toxicity Assessment in a Mouse Model

*L. plantarum* LC5.2, selected for its favorable probiotic properties and absence of adverse traits *in vitro*, was further evaluated for safety in a mouse model. A subacute oral toxicity study showed no signs of toxicity, with a 100% survival rate over 30 days. Mice exhibited no behavioral or physical changes, and body weight analysis revealed no significant differences between the *L. plantarum* LC5.2 and control groups. No local injuries or abnormalities were observed, and daily feed consumption was similar in both groups (Table 6).

### Secretion of Total Secretory Immunoglobulin A

**Fig. 1:**
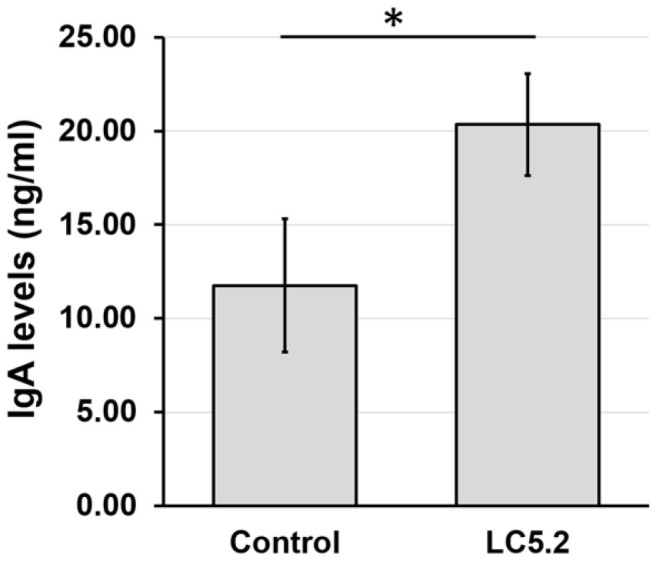
Total secretory IgA levels in the small intestine of mice in the control and *Lactobacillus plantarum* LC5.2 groups. Asterisk (*) indicates a significant difference (*p* < 0.05).

The immunomodulatory effects of *L. plantarum* LC5.2 were assessed by measuring the levels of sIgA in the intestines of mice administered the probiotic, compared to a control group. As illustrated in Fig. 1, the probiotic group showed significantly higher sIgA levels (20.35 ± 2.72 ng/ml) than the control group (11.76 ± 3.56 ng/ml), indicating that *L. plantarum* LC5.2 promotes the production of sIgA in the intestines.

### Pathological Safety Assessment

Histological examination of the small intestine, colon, liver, and spleen revealed no significant histopathological alterations between the control and probiotic-treated groups, suggesting that 30-day probiotic administration did not adversely affect tissue integrity or function.

**Fig. 2:**
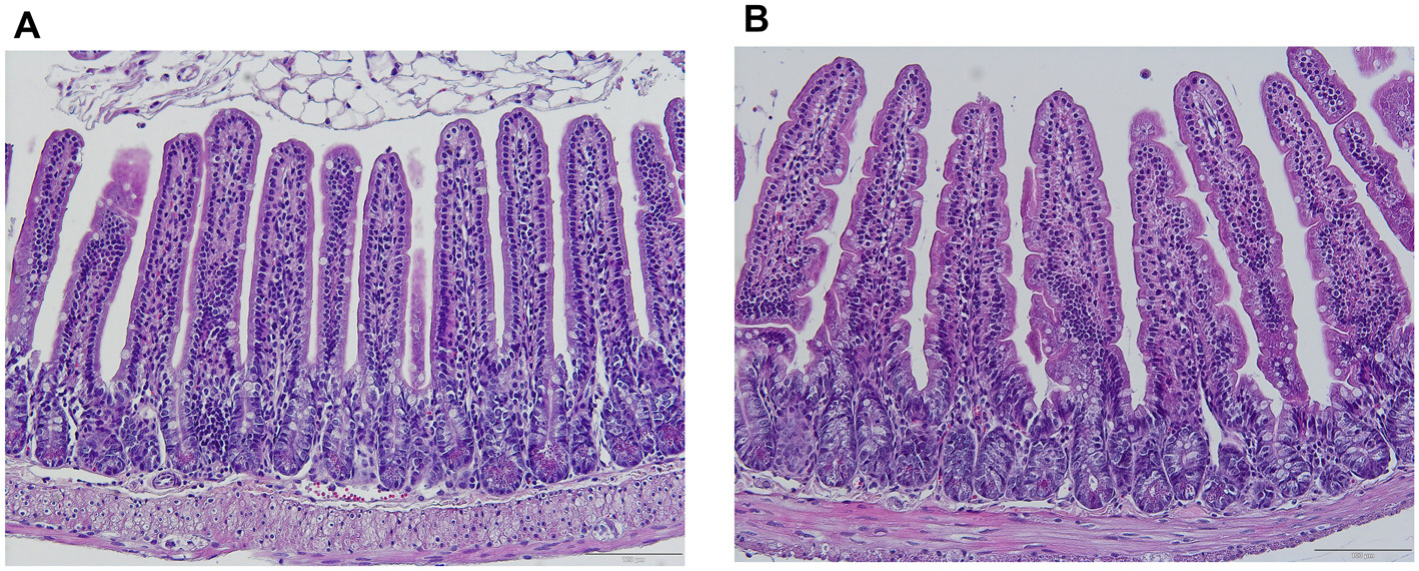
Histological examinations of small intestine in mice. (A) Control and (B) *Lactobacillus plantarum* LC5.2 treatment group. Paraffin-embedded tissues were sectioned and stained with hematoxylin and eosin (H&E) to assess histological changes. Hematoxylin stains cell nuclei blue, and eosin staining cytoplasm and extracellular matrix pink. Sections were viewed at 20× magnification.

In the small intestine, as shown in Fig. 2, both groups exhibited normal tissue architecture, with long villi and intact epithelial linings. Goblet cells were appropriately distributed along the villous surface, and the crypts of Lieberkühn maintained their typical morphology. No epithelial detachment, inflammatory cell infiltration, or tissue necrosis was observed. The submucosa and muscularis externa were structurally preserved, free from edema or pathological changes. These findings indicate that probiotic supplementation did not alter small intestinal morphology.

**Fig. 3:**
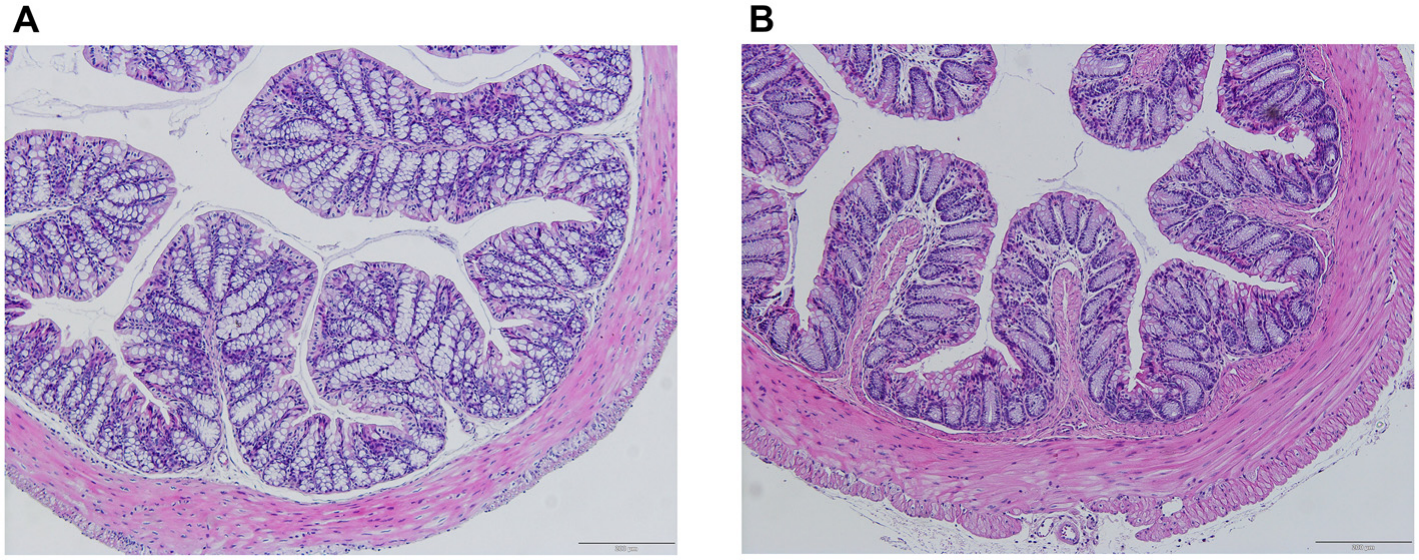
Histological examinations of colon in mice. (A) Control and (B) *Lactobacillus plantarum* LC5.2 treatment group. Paraffin-embedded tissues were sectioned and stained with hematoxylin and eosin (H&E) to assess histological changes. Hematoxylin stains cell nuclei blue, and eosin staining cytoplasm and extracellular matrix pink. Sections were viewed at 20× magnification.

Similarly, histological evaluation of colon tissues revealed normal histoarchitecture, with intact mucosal layers, well-organized crypts of Lieberkühn, and a normal distribution of goblet cells. All structural layers, including the mucosa, submucosa, muscularis externa, and serosa, were preserved, with no signs of epithelial erosion, inflammation, or mucosal thickening. No abnormalities, such as necrosis or crypt distortion, were observed, suggesting that the probiotic did not affect colonic tissue integrity (Fig. 3).

**Fig. 4:**
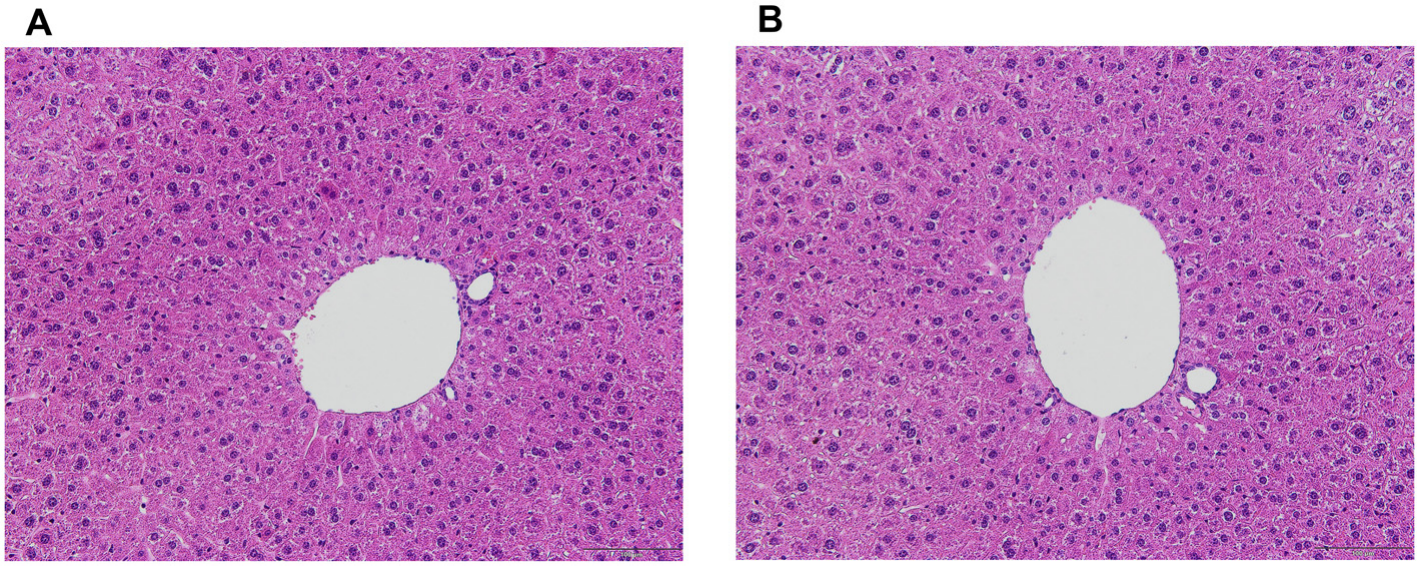
Histological examinations of liver in mice. (A) Control and (B) *Lactobacillus plantarum* LC5.2 treatment group. Paraffin-embedded tissues were sectioned and stained with hematoxylin and eosin (H&E) to assess histological changes. Hematoxylin stains cell nuclei blue, and eosin staining cytoplasm and extracellular matrix pink. Sections were viewed at 20× magnification.

Examination of liver tissues revealed normal hepatic architecture in both groups (Fig. 4). Hepatocytes were organized in cords, with visible sinusoids, and there were no signs of cellular swelling, inflammation, or fatty degeneration. The probiotic-treated group exhibited similar results, with no histopathological abnormalities. Hepatocyte organization remained intact, and there was no evidence of inflammation or tissue damage, indicating that probiotic administration did not induce hepatic toxicity.

**Fig. 5:**
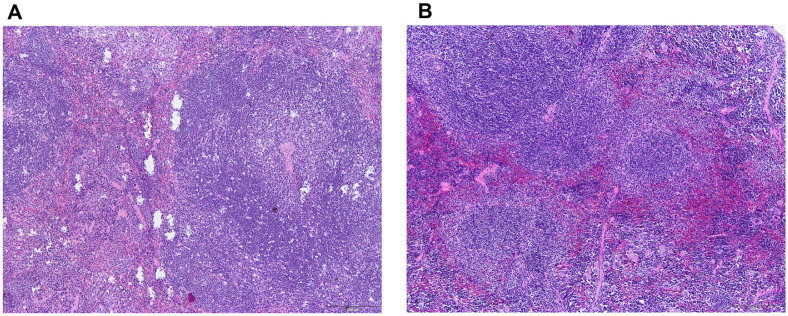
Histological examinations of spleen in mice. (A) Control and (B) *Lactobacillus plantarum* LC5.2 treatment group. Paraffin-embedded tissues were sectioned and stained with hematoxylin and eosin (H&E) to assess histological changes. Hematoxylin stains cell nuclei blue, and eosin staining cytoplasm and extracellular matrix pink. Sections were viewed at 20× magnification.

Lastly, spleen tissues from both groups exhibited normal splenic architecture, with a clear distinction between white pulp and red pulp. The white pulp maintained its normal lymphoid density, and the red pulp showed a typical distribution of vascular sinusoids. No signs of lymphoid depletion, hyperplasia, hemorrhage, or necrosis were observed, confirming that probiotic supplementation did not induce adverse effects on splenic tissue, preserving immune structure integrity (Fig. 5).

### Gut Microbiome Composition

**Fig. 6:**
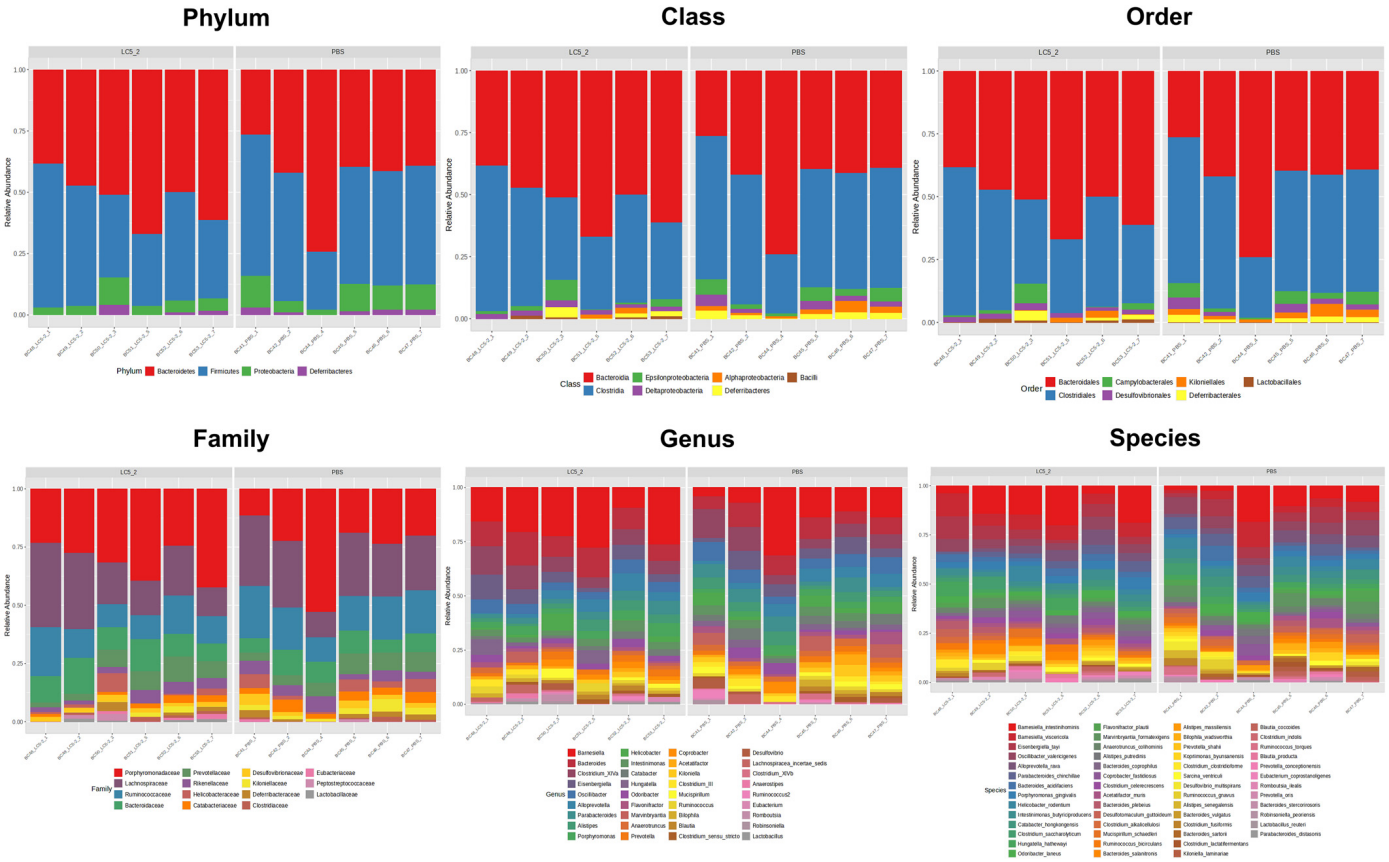
Relative abundance of bacteria at all taxonomic levels in the fecal microbiota of mice administered *Lactobacillus plantarum* LC5.2 compared to the control (PBS) group. Stacked bar chart showing the relative abundance of bacterial taxa at all classification levels (phylum to species) in the fecal microbiota of mice treated with *Lactobacillus plantarum* LC5.2 compared to the PBS control group. Each color represents different bacterial taxa identified in the samples. Taxonomic profiles were generated from 16S rRNA gene sequencing data and the figure was created using MicrobiomeAnalyst.

The 16S rRNA gene of the gut microbiome was analyzed, and the results showed that Good's coverage averaged 100% across all samples, indicating comprehensive sequencing, while rarefaction curves reached saturation, confirming sufficient sequencing depth (data not shown). Taxa abundance analysis at all levels revealed differences in microbial composition between the probiotic and control groups (Fig. 6).

At the phylum level, *Bacteroidetes*, *Firmicutes*, *Proteobacteria*, and *Deferribacteres* were the predominant phyla in both the probiotic and control groups, but their relative abundances differed. The probiotic group exhibited higher *Bacteroidetes* (51.48%) and lower *Firmicutes* (41.19%) compared to the control group, where *Firmicutes* was more dominant (46.40%) with lower *Bacteroidetes* (43.45%).

At the phylum level, the predominant phyla identified were *Bacteroidetes*, *Firmicutes*, *Proteobacteria*, and *Deferribacteres*, all of which were detected in both the probiotic and control groups, though their abundances varied. In the probiotic group, *Bacteroidetes* was the most dominant phylum at 51.48%, followed by *Firmicutes* at 41.19%, *Proteobacteria* at 5.71%, and *Deferribacteres* at 1.62%. In the control group, *Firmicutes* was the most abundant phylum (46.40%), followed by *Bacteroidetes* (43.45%), *Proteobacteria* (8.39%), and *Deferribacteres* (1.76%).

At the class level, *Bacteroidia* (51.48%) and *Clostridia* (40.57%) dominated the probiotic group, whereas the control group exhibited a higher proportion of *Clostridia* (46.40%) and a lower proportion of *Bacteroidia* (43.45%). These trends continued at the order level, where *Bacteroidales* and *Clostridia*les were the most abundant in both groups.

At the family level, the probiotic group showed enrichment in *Porphyromonadaceae* (30.56%), *Lachnospiraceae* (22.61%), *Ruminococcaceae* (13.44%), and *Bacteroidaceae* (10.99%), whereas the control group had higher *Lachnospiraceae* (24.33%), *Porphyromonadaceae* (24.17%), and *Ruminococcaceae* (16.97%).

At the genus level, *Barnesiella*, *Bacteroides*, and *Clostridium_XlVa* were predominant in both groups, but their relative proportions varied. The probiotic group exhibited a higher abundance of *Barnesiella* (19.57%), followed by *Bacteroides* (10.99%) and *Clostridium_XlVa* (8.20%). In contrast, the control group had lower *Barnesiella* (13.19%), *Bacteroides* (8.53%), and *Clostridium_XlVa* (7.46%).

At the species level, the probiotic group contained more *Barnesiella intestinihominis* (12.12%), *Barnesiella viscericola* (7.44%), and *Eisenbergiella tayi* (6.10%), while the control group had *B. intestinihominis* (8.08%), *Oscillibacter valericigenes* (5.79%), and *Eisenbergiella tayi* (5.55%).

**Fig. 7:**
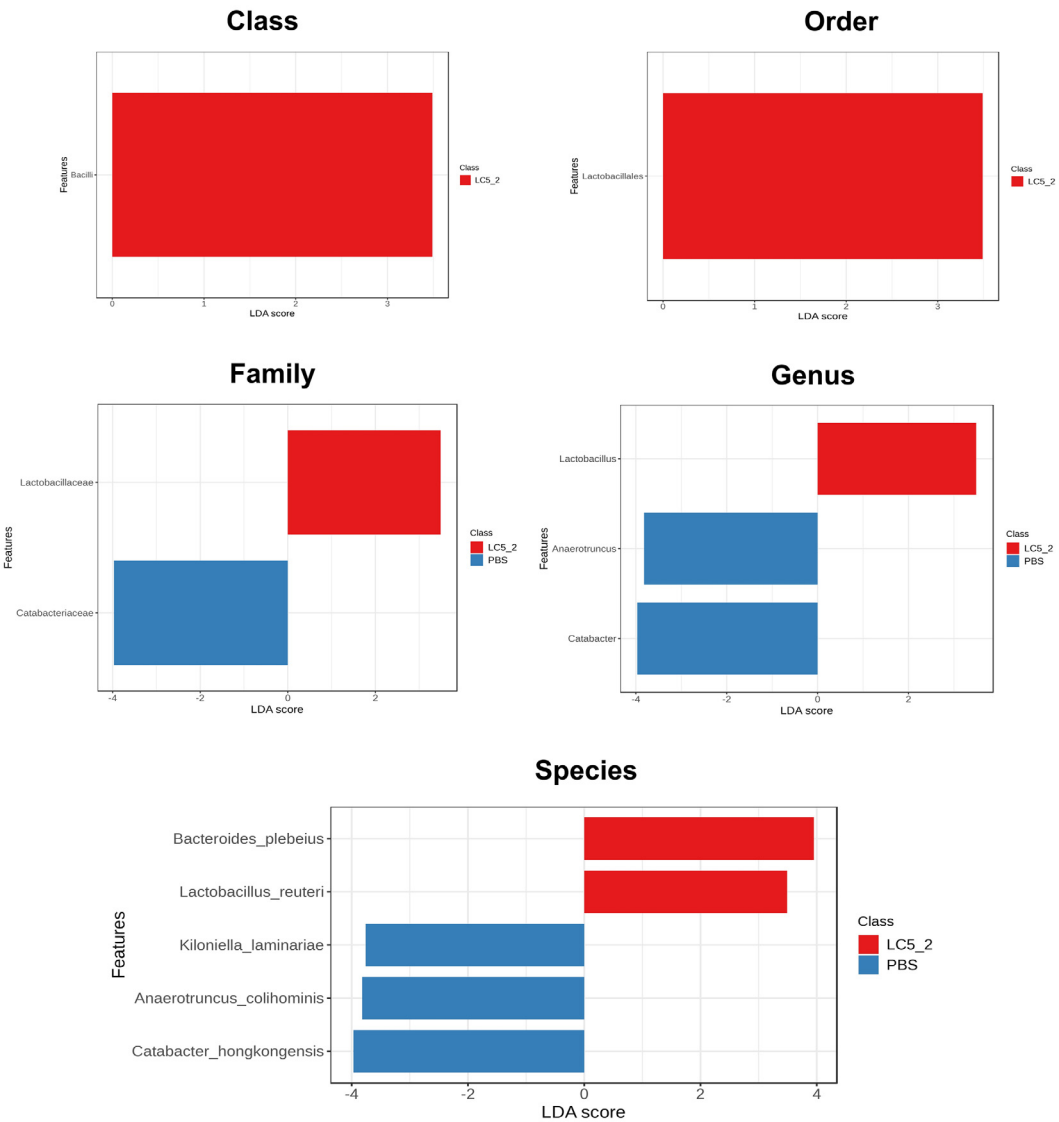
LEfSe comparison of differentially abundant bacterial taxa between mice treated with *Lactobacillus plantarum* LC5.2 and the control group. Blue bars indicate taxa enriched in the control group, red bars in the LC5.2 group, with bar length reflecting the LDA score.

Linear Discriminant Analysis Effect Size (LEfSe) was used to identify taxa with differential abundance between the groups (Fig. 7). The probiotic group showed significant increases in the class *Bacilli* (LDA; 3.49), order *Lactobacillales* (LDA; 3.49), family *Lactobacillaceae* (LDA; 3.49), and genus *Lactobacillus* (LDA; 3.49). At the species level, *L. reuteri* (LDA; 3.49) and *Bacteroides plebeius* (LDA; 3.95) were more abundant in the probiotic group. In contrast, the control group showed increased abundance of *Catabacteriaceae* (LDA; 3.97), *Anaerotruncus* (LDA; 3.82), and *Catabacter* (LDA; 3.97), as well as higher abundance of *Kiloniella laminariae* (LDA; 3.76), *Anaerotruncus colihominis* (LDA; 3.82), and *Catabacter hongkongensis* (LDA; 3.97).

**Fig. 8:**
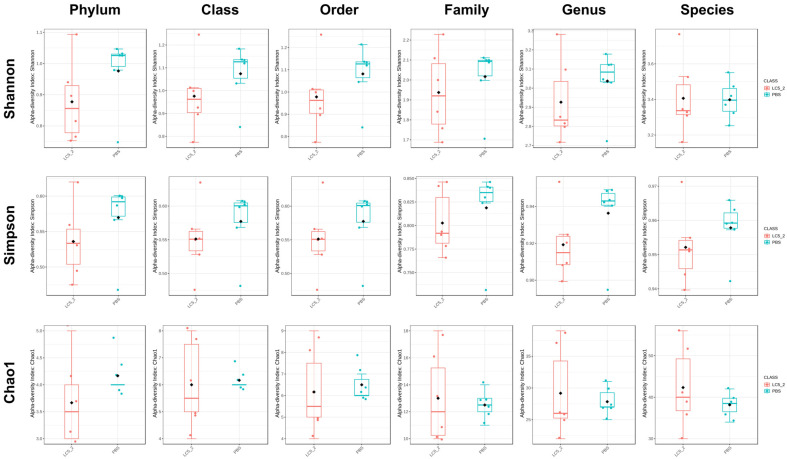
Alpha diversity of the fecal microbiota in *Lactobacillus plantarum* LC5.2-treated and control (PBS) mice, based on Shannon, Simpson, and Chao1 indices. The median is shown by the line inside each box, with whiskers extending to the min and max values. Outliers and individual samples are represented by dots.

**Fig. 9:**
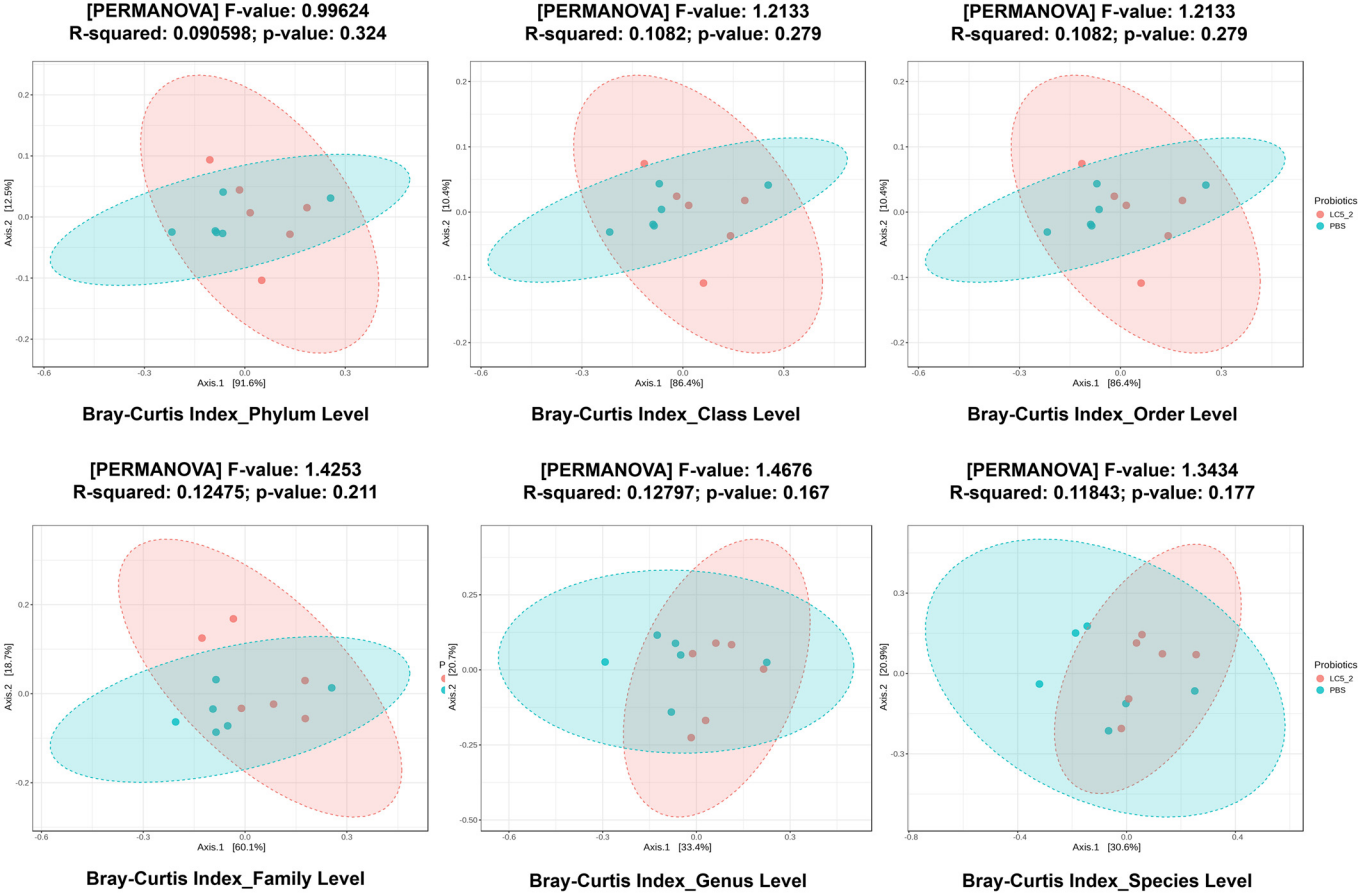
Beta diversity of the microbiome between *Lactobacillus plantarum* LC5.2-treated and control (PBS) mice, based on Bray–Curtis distance. Red dots represent LC5.2, and blue dots represent control.

**Fig. 10:**
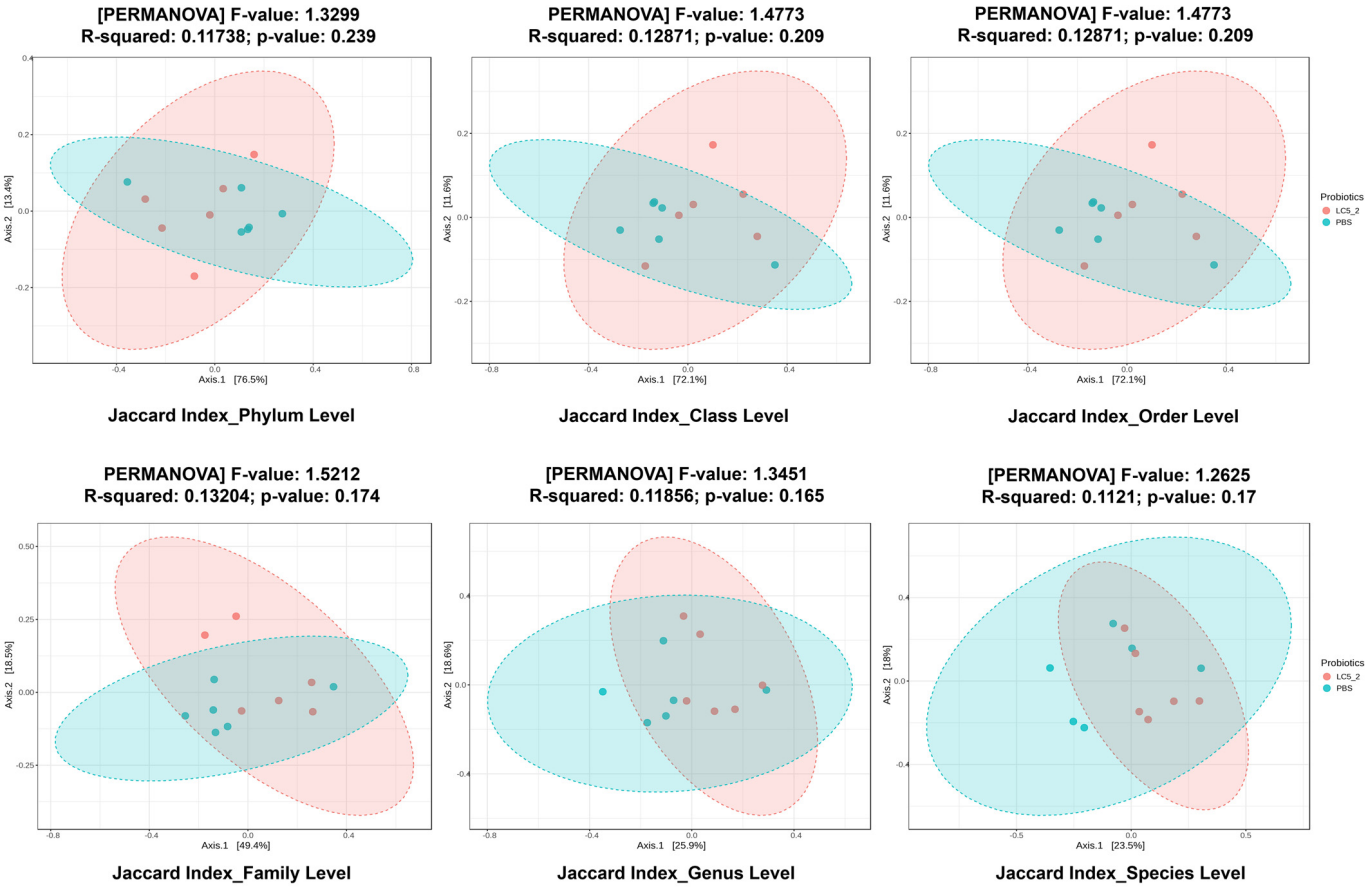
Beta diversity of the microbiome between *Lactobacillus plantarum* LC5.2-treated and control (PBS) mice, based on Jaccard distance. Red dots represent LC5.2, and blue dots represent control.

Alpha diversity, assessed using Shannon, Simpson, and Chao1 indices, showed no significant differences between the groups, as shown in Fig. 8. Similarly, beta diversity analysis, conducted with the Bray-Curtis and Jaccard indices, revealed no significant differences between the groups, as depicted in Figs. 9 and 10. These findings suggest that the probiotic administration did not result in notable changes in the overall diversity of the gut microbiota in the experimental groups.

## Discussion

The gastrointestinal tracts and feces of animals are valuable sources of bacteria for probiotic selection [5, 27]. In this study, native pig feces were selected due to their beneficial characteristics. Native pigs, raised in backyard environments, are frequently exposed to pathogens and are able to overcome them, suggesting a robust and resilient gut microbiota. Their diverse diet, including leftovers, fruit and vegetable scraps, and rice bran, supports a varied gut bacterial community. Additionally, native pigs are less likely to be exposed to antibiotics compared to commercial pigs, which reduces the risk of harboring antibiotic-resistant bacteria. These factors make native pigs an excellent source for identifying beneficial probiotic bacteria while minimizing undesirable traits. In this study, a large number of *Lactobacillus* were isolated from the feces of native pigs, confirming the presence of potential probiotics. *Lactobacillus* strains have also been isolated from the feces of various animals, such as wild boar, native pig and commercial pig [8], dairy cow [28], buffalo calves [29] and poultry [30].

An essential objective of probiotic use in livestock is the prevention of intestinal infections, with antimicrobial activity against pathogens being a key criterion for probiotic selection. Lactic acid bacteria are recognized for their ability to inhibit the growth of foodborne bacterial pathogens through the production of organic acids or bacteriocins [31, 32]. Our study found that a significant number of isolates inhibited EHEC SC2451-1, EPEC SC2451-2, and *S*. Typhimurium SC2451-3. Notably, when the CFCS was neutralized to pH 7, the antibacterial activity was completely lost, suggesting that the inhibition was primarily due to organic acid production [33]. Our results are consistent with previous studies demonstrating that *Lactobacillus* sp. can effectively inhibit pathogens such as EPEC, EHEC, and *S*. Typhimurium [11, 34, 35]. These antibacterial properties are critical for mitigating enteric pathogens responsible for diarrheal diseases in pigs, which significantly impact pig production [36].

After ingestion, probiotic bacteria encounter a low pH in the stomach. Thus, probiotics must survive the acidic environment of the stomach to adhere to and colonize the intestinal tract [6]. In this study, although a large number of *Lactobacillus* isolates with pathogenic inhibition properties were selected, not all isolates were able to withstand low pH conditions. Only 9 isolates met the selection criterion of an acid tolerance rate of 80% or higher. Among them, LC4.2 and LC5.2 demonstrated exceptional acid tolerance, with survival rates of 100% or higher. This suggests that these isolates not only survived but also thrived under acidic conditions, supporting their potential as effective probiotics capable of surviving in the gastrointestinal tract. Previous research indicates that *Lactobacillus* has a doubling time of 1.38 to 2.44 hours [37], highlighting its resilience and adaptability in the gastrointestinal environment, where survival in low pH is essential for colonization and conferring health benefits. This could explain why our *Lactobacillus* isolates exhibited survival rates exceeding 100% at pH 3. Our findings are consistent with the acid resistance mechanisms of *Lactobacillus* species [38], which possess acid resistance systems typical of Gram-positive bacteria [39]. These findings are consistent with studies on the survival of lactic acid bacteria (LAB) in simulated gastric conditions. For instance, it has been demonstrated that twelve LAB strains maintained over 82% survival at pH 3.0 [40], while another study found that ten LAB strains from fermented foods survived over 90% at pH 2.5 for 2 h [41].

Once probiotics have passed through the acidic conditions of the stomach, they are exposed to bile salts in the duodenum, which can interfere with bacterial survival by altering the lipids and fatty acids crucial for maintaining cell membrane integrity. This disruption impacts membrane permeability, viability, and the bacteria’s ability to interact with their environment, making bile resistance critical for their survival [36, 42]. In our study, all nine isolates not only demonstrated remarkable acid tolerance but also exhibited excellent bile tolerance, with survival rates ranging from 89% to 139%. These findings highlight the ability of *Lactobacillus* spp. to adapt to both acidic and bile salt exposures, further reinforcing their potential as effective probiotics. Compared to strains from other environments, our isolates demonstrated strong resilience, supporting their suitability for gastrointestinal survival and potential health benefits [43].

After exhibiting strong acid and bile tolerance, the ability of *Lactobacillus* isolates to adhere to surfaces became a crucial aspect of their potential as probiotics. Adhesion is a complex process that includes both specific and non-specific interactions, such as auto-aggregation and hydrophobicity of the cell surface [44]. Auto-aggregation plays a vital role in helping probiotics colonize and persist in the gastrointestinal system by forming protective barriers that prevent pathogen adhesion [45, 46]. In this study, all nine isolates exhibited strong auto-aggregation, with LC5.2 showing the highest aggregation between 2 to 8 h and maintaining 69.74% at 24 h. These results were significantly higher than those reported for dairy *L. plantarum* strains (28–59%) [14], suggesting potentially superior aggregation abilities, possibly due to specific environmental adaptations. Additionally, these findings align with previous research indicating that auto-aggregation in *Lactobacillus* strains varies depending on incubation time [47].

Following the observation of strong auto-aggregation in the *Lactobacillus* isolates, we further examined the role of cell surface hydrophobicity, which is crucial for the initial interaction between bacteria and host cells, promoting adhesion, a key characteristic of effective probiotics [48]. Strains with higher hydrophobicity generally show better adhesion, enhancing their ability to form protective barriers in the gut [49]. The variability in hydrophobicity observed in this study suggests that certain strains, particularly those with strong hydrophobicity, may be more effective at colonizing the intestinal tract, potentially offering greater probiotic benefits. Additionally, the hydrophobicity of bacterial cell surfaces is influenced by the composition and structure of cell walls and membranes, which include molecules such as teichoic acids, lipoteichoic acids, lipopolysaccharides, and surface proteins such as S-layer [50]. Our results align with previous findings, which reported similar variations in hydrophobicity in *L. plantarum* strains isolated from pig feces (18% to 77%) [11].

In addition to auto-aggregation and cell surface hydrophobicity, we also evaluated the biofilm-forming ability of our *Lactobacillus* isolates. Biofilm formation is an advantageous property for probiotic bacteria, including *Lactobacillus* spp., as it enhances their capacity to colonize and persist on the host’s mucosa, preventing pathogenic bacteria from establishing [51]. All isolates in this study were able to form biofilms, demonstrating moderate adherence, with LC5.2 and LF5.2 exhibiting the highest biofilm-forming abilities. This biofilm formation further supports the probiotic properties of our strains. These findings are consistent with previous research showing that *L. plantarum* can form strong, resilient biofilms *in vitro* [52]. Additionally, *Lactobacillus* spp. from vaginal sources, including *L. crispatus*, *L. gasseri*, *L. vaginalis*, and *L. plantarum*, have been recognized for their biofilm production, with *L. plantarum* noted as a particularly effective biofilm producer [53].

Beyond the properties associated with non-specific interactions, this study also evaluated the adhesion capability via specific interactions by utilizing Caco-2 cells as an intestinal epithelial cell model to assess adhesion related to ligand-receptor interactions. All isolates successfully adhered, with adherence rates ranging from 2.17%to 10.15%. These results align with previous studies, where adhesion rates among probiotic *Lactobacillus* strains ranged from 3–14% [54], while *L. plantarum* from raw milk cheeses showed strain-dependent adhesion of 4.98–14.38% [14]. Interestingly, strains with reported health benefits did not exhibit superior adhesion compared to our isolates, suggesting that Caco-2 adhesion alone may not reliably predict probiotic efficacy *in vivo* [54]. The absence of a mucus layer in Caco-2 cells is a significant limitation [55, 56], which may explain the lower adherence observed in our *Lactobacillus* strains. To better mimic *in vivo* conditions, future studies should consider mucus-producing cell lines such as HT29 [18] or co-culture models to better mimic *in vivo* conditions [57].

Besides antipathogenic activity, co-aggregation is an important mechanism contributing to the antagonistic action against pathogens [58]. It prevents pathogen colonization and accelerates pathogen elimination through faces [6, 59]. Additionally, co-aggregation allows probiotics to produce antimicrobial substances in close proximity to them, which may inhibit the growth of pathogens in the gastrointestinal tract [60]. The high co-aggregation rates observed in this study (62-74% with EHEC, 53-64% with *S*. Typhimurium, and 48-61% with EPEC) underscore the potential of our *Lactobacillus* isolates, particularly LC5.2, to play a vital role in competitive exclusion. This suggests that LC5.2 could be an effective probiotic strain capable of improving gut microbiota balance and reducing pathogen colonization. These findings align with previous studies, which have reported similar co-aggregation capacities in *Lactobacillus* strains [61, 62]. However, the observed differences between isolates highlight the strain-dependent nature of this characteristic, which can vary based on factors such as auto-aggregation properties and incubation duration [47, 63]. This variability emphasizes the need to consider specific strain properties when selecting probiotics for therapeutic or preventive applications.

Alongside the evaluation of functional properties, the safety of probiotics plays an equally crucial role in selecting suitable strains for human and animal applications. The safety of probiotic strains is a fundamental consideration because it directly influences their potential health benefits and overall suitability for use. Given the inherent intraspecies diversity within probiotic species, it is essential to assess both safety and functionality at the strain level, as the Generally Recognized as Safe (GRAS) status of a species cannot be generalized to all its strains. This is particularly important because not all strains exhibit the same safety profile, and safety assessments at the strain level provide a more accurate indication of their suitability for consumption. A key aspect of this safety evaluation is the screening for hemolytic activity, which serves as a predictor of virulence in bacteria. β-hemolysis, indicating the production of hemolysin (an exotoxin that damages red blood cell membranes), may suggest pathogenic potential [64]. On the other hand, γ-hemolysis, which shows no red blood cell lysis, is generally considered a sign of safety. These principles align with recommendations from the Joint FAO/WHO Expert Consultation on the Evaluation of Health and Nutritional Properties of Probiotics in Food, which emphasizes the necessity of hemolysis screening to determine the safety of probiotic strains. In our study, hemolytic activity was assessed using standard microbiological methods, and none of the *Lactobacillus* isolates exhibited β-hemolysis, suggesting the absence of harmful exotoxin production. Most isolates displayed α-hemolysis, a non-pathogenic form often associated with hydrogen peroxide production that results in a greenish coloration of the colony [65]. Notably, LC5.2 exhibited γ-hemolysis, which further supports its safety for use as a probiotic. These findings are consistent with previous research that has also identified α-hemolysis in certain probiotic strains, reinforcing the notion that α-hemolysis is typically non-pathogenic and does not pose a threat to health [64, 66].

Furthermore, while safety is crucial, the functionality of probiotics is equally significant. Previous studies have shown that multi-strain probiotics often offer enhanced benefits for animal health due to synergistic interactions between strains, which can improve their effectiveness in promoting gut health and preventing pathogen colonization [67-72]. In this study, we assessed the antagonistic interactions between *Lactobacillus* isolates to explore their potential for use in multi-strain probiotic formulations. Remarkably, no antagonistic interactions were observed, indicating that these isolates can function effectively both as single-strain and multi-strain probiotics. This finding suggests that these *Lactobacillus* strains could be used in combination for greater probiotic efficacy, enhancing their potential for animal health applications.

Following hemolytic activity testing, antibiotic susceptibility testing provides additional insight into the overall safety of probiotic strains. According to FAO/WHO guidelines, probiotics should not harbor transferable antibiotic resistance genes, as these may facilitate horizontal gene transfer to pathogens and pose public health risks. Because probiotics are usually ingested in large amounts and closely interact with the host’s gut microbiota, their potential to transfer resistance genes within the gastrointestinal tract, which has a high microbial density and frequent cell-to-cell contact, requires careful consideration [73]. However, the evaluation of antibiotic resistance in *Lactobacillus* is complicated by the absence of standardized methods and defined susceptibility breakpoints for many antibiotics. In this study, all *Lactobacillus* isolates exhibited resistance to vancomycin and tetracycline. These findings align with previous reports indicating intrinsic resistance to vancomycin among *Lactobacillus* species [74, 75], and widespread tetracycline resistance, particularly in dairy-derived strains [76-78]. Furthermore, previous study also reported resistance to several antibiotics in commercially available probiotic *Lactobacillus* strains [79]. Importantly, this resistance is generally intrinsic and not linked to transferable mechanisms, suggesting minimal safety concerns. Intrinsic resistance does not typically contribute to the spread of resistance genes. Moreover, such resistance may benefit probiotics by allowing them to persist during antibiotic therapy, maintaining their positive effects on gut health. Nonetheless, ongoing monitoring remains essential to ensure these strains do not acquire or transmit transferable resistance genes that could compromise their safety.

Accurate strain identification plays a pivotal role in ensuring the safety and functionality of probiotic candidates. The confirmation that all selected isolates belong to *L. plantarum* highlights not only the genetic consistency among the screened strains but also the effectiveness of a stepwise identification approach. While initial 16S rRNA analysis suggested close similarity to *L. plantarum* and *L. pentosus*, this level of resolution was insufficient due to the high genetic homology within the *L. plantarum* group [80]. The use of species-specific recA-based PCR allowed for more definitive classification, demonstrating the value of targeted molecular tools in distinguishing closely related taxa [22]. The convergence of all isolates to *L. plantarum* does not reflect a lack of microbial diversity in the native pig microbiota, but rather points to a targeted selection strategy based on desirable probiotic traits. This result supports the idea that certain species, such as *L. plantarum*, may be more dominant or functionally robust under the specific screening criteria used. Furthermore, it underscores the relevance of using multiple identification methods to avoid misclassification, which could compromise both the safety profile and the intended health claims of a probiotic product. In this context, accurate species- and strain-level identification is not merely a taxonomic exercise, but a critical step in ensuring traceability, reproducibility, and regulatory compliance.

The *in vitro* evaluation of *L. plantarum* LC5.2 revealed promising probiotic characteristics, with the strain exhibiting beneficial properties and lacking any undesirable traits. This initial finding suggests that LC5.2 possesses the essential features of a safe and functional probiotic. To validate these observations under *in vivo* conditions, a subacute oral toxicity study was conducted in a mouse model, providing further insight into its safety profile. Over a 30-day administration period, the mice receiving LC5.2 showed no clinical signs of toxicity, behavioral abnormalities, or any significant physical changes. These results not only reinforce the strain’s safety but also indicate that LC5.2 can maintain its probiotic functions without inducing harmful effects on the host. Moreover, the absence of adverse outcomes in our study agrees with previous research on the safety of *Lactobacillus* strains, such as *L. casei* IMV B-7280 [81] and *L. fermentum* MTCC-5898 in mice [82], which similarly exhibited no toxicity in mouse models. This consistency across different strains and study designs highlights the reliability of *L. plantarum* LC5.2 as a safe candidate for further probiotic development.

The immunomodulatory capacity of *L. plantarum* LC5.2 was further confirmed through its ability to enhance intestinal secretory immunoglobulin A production. Oral administration of LC5.2 resulted in a significant increase in sIgA levels, indicating that the strain effectively stimulates mucosal immune responses. As sIgA is a critical component of mucosal immunity, its elevation suggests that LC5.2 could play an important role in enhancing the intestinal defense mechanisms, including preventing pathogen colonization, maintaining epithelial barrier integrity, and modulating the gut microbiota composition [83]. These findings are consistent with previous studies where similar effects were observed following the administration of other probiotic strains, such as *L. rhamnosus* CCFM1228, 28L2, and W6L1 [84], *Bifidobacterium bifidum* FL228.1 and FL276.1[85], *B. animalis* ssp. *lactis* HY8002 [86], and *L. plantarum* 19-2 [87]. Collectively, these results further substantiate the immuno-modulatory potential of LC5.2, reinforcing its suitability for development as a functional probiotic aimed at enhancing gut immune health.

Histological analysis is an essential method for evaluating the safety of probiotic strains, providing critical information on their effects on tissue integrity and the absence of pathological changes in various organs. In this study, histological examination of the small intestine and colon showed that *L. plantarum* LC5.2 does not disrupt gut integrity or cause tissue damage, further confirming its non-toxic nature and supporting its safety for consumption. Additionally, the liver and spleen were assessed for bacterial translocation, where live bacteria might migrate from the gastrointestinal tract to other organs [88]. No evidence of translocation was observed, indicating that LC5.2 does not possess invasive properties. This finding strengthens the strain’s safety profile, suggesting that it does not invade internal tissues or lead to systemic infections. These results align with previous studies demonstrating that *L. casei* IMV B-7280 [81], *L. buchneri* FD2, and *L. fermentum* HM3 [23] do not cause tissue damage or bacterial translocation. These findings collectively confirm that well-chosen probiotic strains do not pose a risk of tissue damage or bacterial dissemination when taken orally.

Microbial communities play a crucial role in host physiology, influencing nutrient metabolism, immune function, and overall health [89]. In this study, the administration of *L. plantarum* LC5.2 had a notable impact on gut microbiota composition, which highlights its potential to modulate gut health. The observed shift in microbial balance, with increased *Bacteroidetes*, decreased *Firmicutes*, and reduced *Proteobacteria* (which includes several pathogens), suggests that *L. plantarum* LC5.2 may promote a microbial environment that supports optimal metabolic and immune functions while helping to reduce bacteria potentially associated with disease pathogenesis. This shift aligns with previous findings, where *Bacteroidetes* were associated with fiber degradation and SCFA production, processes important for gut health [90-93]. Moreover, this shift was reflected at all taxonomical levels. Notably, the genus *Barnesiella*, which showed higher abundance in the probiotic group, is recognized for its role in gut health, particularly in the production of volatile fatty acids and bile acids that aid in fatty acid absorption [94]. Additionally, *Limosilactobacillus reuteri* was detected exclusively in the probiotic group, suggesting that LC5.2 may promote the growth of beneficial *Lactobacillus* species. *L. reuteri* is known for its metabolic mechanisms that enhance the production of anti-inflammatory cytokines, modulate gut microbiota, and produce antimicrobial molecules like reuterin, which help inhibit pathogens and modulate the immune system [95]. Its presence reinforces the beneficial effects of probiotic supplementation on gut health. These results underscore the potential of *L. plantarum* LC5.2 to modulate the gut microbiota favorably, contributing to a healthier gut environment. Linear Discriminant Analysis Effect Size (LEfSe) confirmed significant increases in *L. reuteri* and *Bacteroides plebeius*. Conversely, the control group showed higher abundances of taxa linked to pathogenicity, such as *Anaerotruncus colihominis* and *Catabacter hongkongensis* [96, 97], indicating a potentially unfavorable gut microbiome. Despite these differences in taxa abundance, no significant changes in alpha or beta diversity were observed, indicating that the overall microbial diversity and community structure remained stable. These results highlight the role of probiotics in selectively enriching beneficial microbiota while maintaining overall microbial balance, which is critical for gut health.

Overall, this study identified *L. plantarum* LC5.2 as a promising probiotic strain with significant potential for use in livestock. *In vitro*, the strain demonstrated excellent tolerance to acidic and bile conditions, strong adhesion properties, anti-pathogenic activity, and safety for use in probiotic formulations. *In vivo*, *L. plantarum* LC5.2 showed no toxicity in a mouse model, enhanced gut immune function, and beneficial effects on gut microbiome composition. These findings establish the strain as a robust probiotic candidate, with future research needed to confirm its long-term impact on livestock health and productivity.

## Figures and Tables

**Table 1:** Acid and bile tolerance of nine *Lactobacillus plantarum* strains from native swine feces.

Strains	Acid tolerance (%)	Bile tolerance (%)
LC 1.1	87.32 ± 3.32^c^	97.81 ± 1.89^d^
LC 4.2	113.76 ± 2.85^a^	139.01 ± 1.71^a^
LC 5.1	80.19 ± 5.77^c^	111.50 ± 2.02^c^
LC 5.2	100.00 ± 0.00^b^	100.00 ± 0.00^d^
LC 5.6	88.71 ± 3.28^c^	115.18 ± 4.29^b^
LF 5.2	82.38 ± 3.84^c^	92.36 ± 2.41^d^
LF 5.3	86.55 ± 1.46^c^	89.73 ± 3.56^d^
LS 5.6	80.00 ± 0.00^c^	98.41 ± 2.75^d^
LS 8.7	90.40 ± 4.22^c^	90.15 ± 5.23^d^

Values represent the mean ± SD from three independent measurements (*n* = 3) for each sample. Different superscripts within the same column indicate a significant difference (*p* < 0.05).

**Table 2:** Adhesion properties of nine *Lactobacillus plantarum* strains from native swine feces.

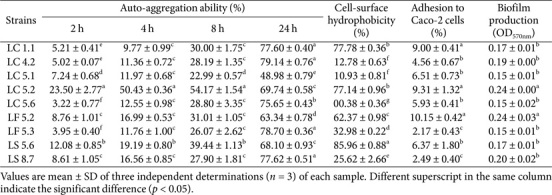

**Table 3:** Co-aggregation with pathogens of nine *Lactobacillus plantarum* from native swine feces.

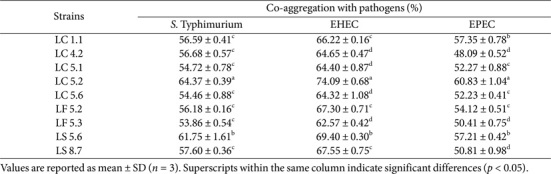

**Table 4:** Safety profiles of nine *Lactobacillus plantarum* from native swine feces.

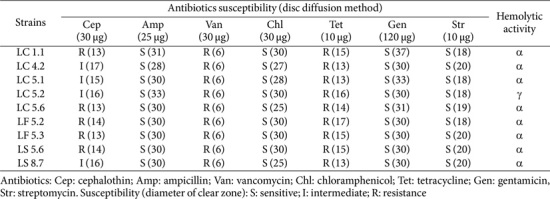

**Table 5:** Species-level identification of *Lactobacillus* by 16S rRNA gene and recA gene.

Strains	Molecular identification		Accession number by NCBI	Biological deposit number by TBRC
	16s rRNA gene	*recA* gene		
LC 1.1	*L. plantarum*	*L. plantarum*	MZ208268	TBRC 15414
LC 4.2	*L. plantarum*	*L. plantarum*	MZ208271	TBRC 15418
LC 5.1	*L. plantarum*	*L. plantarum*	MZ208272	TBRC 15419
LC 5.2	*L. pentosus*	*L. plantarum*	MZ208273	TBRC 15420
LC 5.6	*L. plantarum*	*L. plantarum*	MZ208274	TBRC 15421
LF 5.2	*L. plantarum*	*L. plantarum*	MZ208277	TBRC 15423
LF 5.3	*L. plantarum*	*L. plantarum*	MZ208278	TBRC 15424
LS 5.6	*L. plantarum*	*L. plantarum*	MZ208280	TBRC 15427
LS 8.7	*L. plantarum*	*L. plantarum*	MZ208281	TBRC 15428

**Table 6:** General observations from oral toxicity study of *Lactobacillus plantarum* LC5.2 in mouse model.